# Engineering Genome‐Free Bacterial Cells for Effective SARS‐COV‐2 Neutralisation

**DOI:** 10.1111/1751-7915.70109

**Published:** 2025-03-05

**Authors:** Yutong Yin, Chang Liu, Xianglin Ji, Yun Wang, Juthathip Mongkolsapaya, Gavin R. Screaton, Zhanfeng Cui, Wei E. Huang

**Affiliations:** ^1^ Department of Engineering Science University of Oxford Oxford UK; ^2^ Wellcome Centre for Human Genetics, Nuffield Department of Medicine University of Oxford Oxford UK; ^3^ Chinese Academy of Medical Science (CAMS) Oxford Institute (COI) University of Oxford Oxford UK; ^4^ Oxford Suzhou Centre for Advanced Research (OSCAR) University of Oxford, Suzhou Industrial Park Suzhou Jiangsu China; ^5^ Mahidol‐Oxford Tropical Medicine Research Unit Bangkok Thailand; ^6^ Department of Medicine University of Oxford Oxford UK; ^7^ Institute of Biomedical Engineering, Department of Engineering Science University of Oxford Oxford UK

**Keywords:** bacterial therapy, chromosome‐free, live virus neutralisation, mini‐SimCell, SimCell, synthetic biology, synthetic cell

## Abstract

The COVID‐19 pandemic has caused unparalleled impacts on global social dynamics, healthcare systems and economies, highlighting the urgent need for effective interventions to address current challenges and future pandemic preparedness. This study introduces a novel virus neutralisation platform based on SimCells (~1 μm) and mini‐SimCells (100–200 nm), which are chromosome‐free and non‐replicating bacteria from an LPS‐free *Escherichia coli* strain (ClearColi). SimCells and mini‐SimCells were engineered to display nanobodies on their surface, specifically targeting the receptor‐binding domain (RBD) of the SARS‐CoV‐2 spike protein – a critical immunogenic fragment essential for viral entry into host cells. It was demonstrated that nanobody‐expressing SimCells achieved over 90% blocking efficiency against synthesised RBD from both the original Wuhan and the B.1.351 (Beta) variant using competitive enzyme‐linked immunosorbent assay (ELISA) assay. More importantly, live virus neutralisation assays demonstrated that NB6 nanobody‐presenting mini‐SimCells effectively neutralised the live SARS‐CoV‐2 Victoria variant with an IC50 of 2.95 × 10^9^ ± 1.40 × 10^8^ mini‐SimCells/mL. Similarly, VE nanobody‐presenting mini‐SimCells effectively neutralised the B.1.351 (Beta) variant of the SARS‐CoV‐2 virus with an IC50 of 5.68 × 10^9^ ± 9.94 × 10^8^ mini‐SimCells/mL. The mini‐SimCells successfully protected Vero cells, a cell line derived from the kidney of an African green monkey, from infection by the live virus of SARS‐CoV‐2 and its variants. These results suggest that SimCell‐based neutralisation offers a promising strategy for the prevention and treatment of SARS‐CoV‐2, and potentially other viral infections.

## Graphical abstract

Neutralisation of virus using mini‐SimCells.

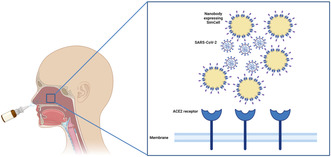

## 1 Introduction

In 2020, the entire world transitioned into pandemic mode, profoundly altering everyone's life due to the emergence of a novel coronavirus, SARS‐CoV‐2 (severe acute respiratory syndrome coronavirus 2) (Kumar et al. 2021). This period witnessed the fastest vaccine development and distribution in history, driven by an extensive global collaboration. As we move forward, it has become increasingly clear that SARS‐CoV‐2 will become an endemic virus, meaning we will likely live with it (Raman et al. 2021). Although vaccines have been pivotal in mitigating the virus's most severe impacts, the ongoing emergence of variants and breakthrough infections highlight the critical need for innovative strategies to address SARS‐CoV‐2, as well as to prepare for future viral pandemics. This pinpoints the urgent requirement for the development of effective prophylactics and therapeutic interventions to combat not only the current pandemic but also future outbreaks.

A few monoclonal antibodies, such as sotrovimab, bebtelovimab and ronapreve, have been approved for effectively neutralise SARS‐CoV‐2 and used to treat the infection (Cox et al. 2022). The cost of the antibody drugs ranges from £1000 to £2000 per treatment course. Unlike the chemical drugs available in pharmacies, monoclonal antibodies are complex and expensive to produce. Nanobodies (Nbs), which are the variable domains (VHH) of heavy‐chain‐only antibodies derived from camelids (Hamers‐Casterman et al. 1993), exhibit greater stability and reduced immunogenicity compared to full‐length antibody sequences, making them more suitable for human administration (Güttler et al. 2021). At only 15 kDa, nanobodies offer enhanced potency, better recognition capabilities and the ability to bind to epitopes that are inaccessible to traditional antibodies (Xu et al. 2021). Their capacity for engineering into multivalent forms allows for the simultaneous binding to multiple target sites, significantly boosting their effectiveness in neutralising viruses and mitigating the risk of mutational escape by variants (Koenig et al. 2021). Nanobodies can be tailored to specifically target and neutralise active sites of viruses, offering more effective neutralisation than conventional antibodies. Therefore, they present a promising alternative to monoclonal antibodies for the treatment of SARS‐CoV‐2 symptoms.

Research suggests that for intranasal delivery, particle sizes ranging from 10 to 200 nm are deemed optimal. Smaller particles, specifically those between 10 and 100 nm, are favoured for their ability to penetrate mucosal barriers more effectively and their reduced likelihood of being cleared rapidly by mucociliary mechanisms (Lai et al. 2009; Schuster et al. 2013). This preference is supported by evidence demonstrating that nanovesicles, such as liposomal‐based nanoparticles and exosomes (~100 nm), when adorned with SARS‐CoV‐2 neutralising antibodies, are 10 times more efficient than the antibodies alone (Chen et al. 2021). This efficiency highlights the role of nanoparticles in enhancing antibody stability and, consequently, the effectiveness of virus capture and clearance, presenting a potential therapeutic avenue for SARS‐CoV‐2 infections (Cocozza et al. 2020; Zhang et al. 2020; Chen et al. 2021; Li et al. 2021; Wang et al. 2021; El‐Shennawy et al. 2022). Conversely, a study using microvesicles ~1.26 μm in size revealed that both nanovesicles and the solid core of membrane‐coated particles, such as polymeric particles coated with an ACE2 membrane, failed to completely isolate the virus from host cells (Hu et al. 2023). Despite the demonstrated promise in virus neutralisation, most approaches involve adorning particle surfaces with angiotensin‐converting enzyme 2 (ACE2) receptors as a decoy strategy for neutralising SARS‐CoV‐2 variants. However, the interaction of ACE2 receptors with host cellular processes could have unforeseen effects on the host's health, warranting further investigation.

To neutralise SARS‐CoV‐2 fusing to the host cell, we targeted the receptor‐binding domain (RBD) region of spike protein, a critical region that allows the virus to gain entry to the host cell. Studies identified three classes of SARS‐CoV‐2 neutralising nanobodies (Sun et al. 2021; Xu et al. 2021): Class 1 nanobodies physically block the binding between spike protein and hACE‐2 receptor with competitive binding. Class 2 nanobodies recognise a highly conserved epitope on RBD, which is often inaccessible by conventional antibodies. Class 3 nanobodies stabilise spike protein in the post‐fusion conformation, preventing binding to the hACE2. In this study, we developed the modular nanobody display platform (Salema and Fernández 2017) to readily express RBD‐neutralising nanobodies on the surface of the SimCell chassis. We selected four nanobody candidates: TY1 (Hanke et al. 2020), NIH‐CoV2nb‐112 (Esparza et al. 2020), mNb6 (Schoof et al. 2020) and bivalent nanobody VE (Koenig et al. 2021). TY1 is a monomeric nanobody isolated from immunised Alpaca, which showed high affinity towards the SARS‐CoV‐2 RBD (Hanke et al. 2020). TY1 competitively binds to SARS‐CoV‐2, physically hindering the interaction between the virus and the human receptor. NIH‐CoVnb‐112 (Esparza et al. 2020) from the phage display library exhibited competitive binding to RBD and showed high neutralisation efficiency against pseudo‐typed lentivirus. mNB6 is a monomeric nanobody isolated from the yeast display library, which showed high sensitivity and efficient neutralisation at a low nanomolar range (Schoof et al. 2020). Both mNb6 and TY1 can stabilise spikes in the post‐fusion form, preventing the interaction between spike protein and hACE2 from cell fusion. Lastly, bivalent nanobody VE consists of nanobody V and nanobody E joint by a neutral protein linker (Koenig et al. 2021). V and E each bind to a distinct epitope on RBD, one diverse and one conserved. VE showed promising results for neutralising SARS‐CoV‐2 variants and simultaneously targeting two epitopes, effectively preventing the emergence of mutational escape.

In our research, we developed LPS‐free and non‐replicating bacterial chassis, SimCells (1–2 μm) and mini‐SimCells (100–200 nm), as safe carriers for displaying anti‐SARS‐CoV‐2 RBD nanobodies in a ‘plug‐and‐play’ manner (Lim et al. 2022; Mamat et al. 2013; Fan et al. 2020). We initially verified the blocking efficiency of nanobody‐expressing SimCells through an in vitro blocking assay. We then validated the neutralisation efficiency of mini‐SimCells against live Victoria and Beta variants using a live virus neutralisation assay. Our aim was to explore the therapeutic potential of nanobodies in conjunction with the biological benefits of SimCells to forge a highly effective, stable, cost‐efficient and safe ‘smart particle’. This innovative strategy holds promise as both a preventive and neutralising agent, potentially applicable not only to SARS‐CoV‐2 infections but also to other viral infectious diseases, making it a viable candidate for clinical translation.

## 2 Results

### 2.1 Confirmation of Nanobody Surface Display on *Escherichia coli* ClearColi

**FIGURE 1:**
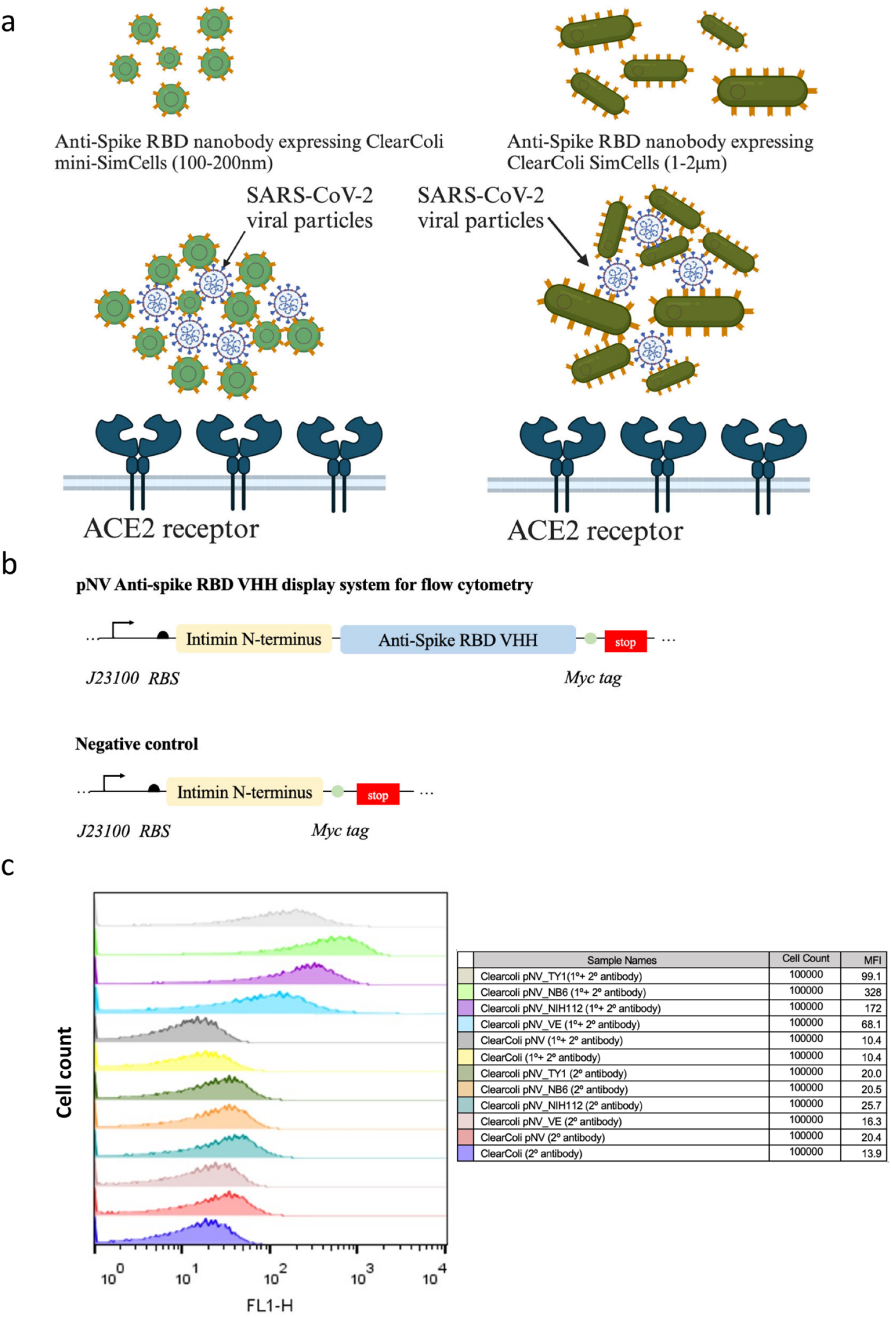
(a) A schematic illustration demonstrating nanobody‐expressing SimCells and mini‐SimCells capturing SARS‐CoV‐2 viral particles, thereby blocking the interaction between the spike RBD and human ACE2 to prevent infection. (b) Architectures of constructs utilised in the flow cytometry experiment: pNV_VHH and pNV_negative control. The Nanobody display system includes Intimin as the outer membrane anchor and VHH as the nanobody used in this study, encompassing TY1, NIH‐CoVnb‐112 and the bispecific Nb6 VE for SARS‐CoV‐2 RBD binding. J23100 regulates the expression of the nanobody display system, whilst Myc serves as the tag for flow cytometry analysis. (c) Histograms from flow cytometry of *E. coli* ClearColi DE3 carrying the pNV nanobody display system. Samples were treated with both primary anti‐Myc antibody and secondary Alexa Fluor 488 antibody. The X‐axis represents the FL1 channel (Alexa Fluor 488 antibody) in relation to the cell count on the Y‐axis. Untransformed ClearColi DE3 and ClearColi transformed with the negative control plasmid pNV_ served as controls. Fluorescent values are reported as median fluorescence intensity (MFI).

The specific LPS‐free *E. coli* mutant ClearColi has been chosen to express the surface display of nanobodies, because LPS‐free ClearColi avoids the unnecessary stimulation of TCR in the immunity system, and the smooth surface of ClearColi enables the small nanobody (~2 nm) exposed to the targeted antigen. We have adopted the pNV (Lim et al. 2022; Salema and Fernández 2017) surface display system driven by a strong promoter J23100 (http://parts.igem.org/Catalog) to express SARS‐CoV‐2 RBD binding nanobodies, TY1, NIH‐112, NB6 and a bivalent nanobody VE (Figure 1a). We constructed all plasmids using NEB HiFi assembly, and we used immunofluorescence followed by flow cytometry to confirm the surface display of nanobodies on *E. coli* ClearColi. For immunofluorescence tagging, we used a primary anti‐My‐c tag antibody to bind Myc‐tagged nanobodies, followed by the detection using a secondary Alexa Fluor 488 conjugated antibody to produce a fluorescence signal. The immunofluorescence samples were analysed using a fluorescent flow cytometer FACS (Figure 1b), which showed stronger fluorescence signals in the nanobody expressing *E. coli* ClearColi (DE3) strains: pNV_TY1, pNV_NB6, pNV_NIH112 and pNV_VE (Table S1) compared to the control of ClearColi (DE3) WT. The results suggest that the nanobodies should have been successfully displayed on the surface of ClearColi.

### 2.2 Nanobodies Displaying on *E. coli* ClearColi Binding to SARS‐CoV‐2 RBD

**FIGURE 2:**
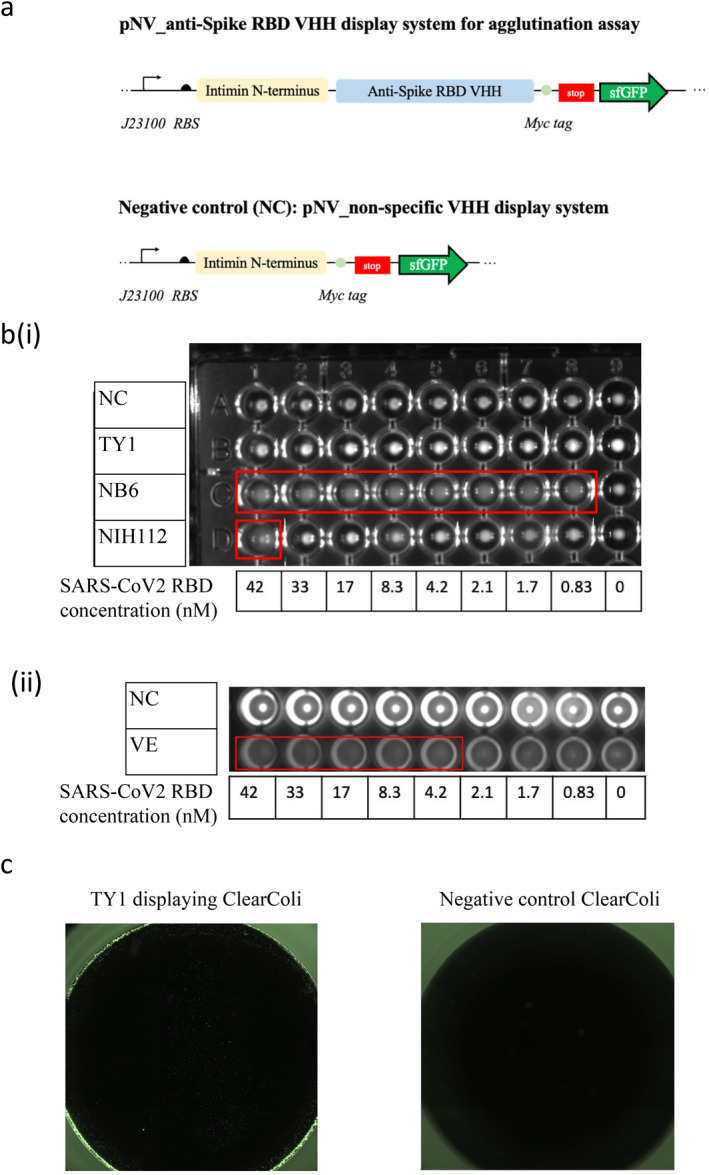
(a) pNV_non‐specific VHH as a negative control. The nanobody display system includes Intimin as the outer membrane anchor and VHH as the nanobody, featuring TY1, NIH‐CoVnb‐112 and the bispecific Nb6 VE for SARS‐CoV‐2 RBD binding. Nanobody 2Rs15d, targeting human epidermal growth factor receptor 2 (HER2), served as the non‐specific control. J23100 controls the expression of the nanobody display system, with sfGFP acting as the fluorescence reporter for imaging. (b) Cell agglutination test of nanobody‐expressing strains: (i) TY1, NB6 and NIH112; (ii) VE; with pNV_antiHER2 VHH_sfGFP serving as the negative control (NC). Positive binding between the displayed nanobodies and the targeted antigen (SARS‐CoV‐2 RBD) results in a cloudy cell suspension, whereas no binding results in a cell pellet. Red boxes indicate positive agglutination (cloudy cell suspension). Images were captured using the VersaDoc Imaging System under the FITC channel. (c) 42 nM of SARS‐CoV‐2 RBD was affixed to the bottom of the well. OD600 = 1 washed pNV_TY1_sfGFP, and pNV_antiHER2_sfGFP (NC) were added to the well and incubated for 1 h at room temperature. Images were taken with a BioTek Cytation5 imaging reader using the excitation/emission wavelength of 488/530 nm for sfGFP detection. Cell adhesion was observed with RBD‐targeting pNV_TY1_sfGFP only; the NC did not exhibit any cell adhesion to the SARS‐CoV‐2 RBD‐coated microplate.

Next, we conducted a cell agglutination test to confirm ClearColi expressing nanobodies binding to the SARS‐CoV‐2 RBD (Table S2) and to evaluate the multivalency of the nanobodies. This cell agglutination test, serving as a biological analogue to the well‐established in vitro latex agglutination test, involves the formation of cross‐links between multivalent nanobodies displaying on the surface of bacterial cells and the target antigen (Lim et al. 2022; Salema and Fernández 2017). This interaction results in cell agglutination and shows a visible cloudy suspension. In contrast, in the absence of the target antigen, the cells settle at the bottom of the well, forming a pellet. A GFP reporter gene was cloned into all pNV_constructs at this stage for better visualisation (shown as pNV_sfGFP, Figure 2a). We selected the RBD antigen concentration ranging from 0.83 to 42 nM. For the negative control, we used the irrelevant anti‐HER2 nanobody, 2Rs15d, known for its specific binding to human epidermal growth factor receptor 2 (HER2) (Pruszynski et al. 2019; Ducharme et al. 2023) which should not bind to the RBD antigen. The antigens were mixed with an equal concentration of cell cultures in a round bottom well plate and incubated the plate overnight at room temperature. We observed agglutination across all concentrations with pNVNB6_sfGFP, whilst pNV_NIH112_sfGFP only exhibited binding at the highest concentration of 42 nM RBD. The pNVTY1_sfGFP and the negative control pNV_antiHER2_sfGFP showed no agglutination for all of the RBD concentrations (Figure 2b). The results demonstrated the selective RBD binding function of NB6 and NIH112, with NB6 detecting antigens at the lower molar concentrations. Hence, we concluded that pNVNB6_sfGFP had the highest sensitivity targeting against SARS‐CoV‐2 RBD, confirming the findings reported in the original paper (Schoof et al. 2020). We used flow cytometry to verify the surface expression of a 30 kDa bispecific nanobody VE, composed of both nanobody V and nanobody E conjugated by a neutral protein linker (Figure 1c). In the cell agglutination assay, pNV_VE sfGFP could detect RBD antigens up to 4.2 nM (Figure 2b). To further validate the binding of pNVTY1_sfGFP to SARS‐CoV‐2 RBD, we performed whole‐cell ELISA by fixing the S1 RBD antigen onto the bottom of microplates and incubating pNVTY1_sfGFP in the RBD coated plate. Fluorescence microscopic images captured by BioTek Cytation5 imaging reader showed that only pNVTY1_sfGFP expressing cells could adhere onto the SARS‐CoV‐2 RBD coated plate, whilst the negative control pNV antiHER2_sfGFP cells were washed away, unable to bind to the plate (Figure 2c). This further underscored the specific binding of pNVTY1_sfGFP to its target RBD. This result also suggests that the surface display of TY1 nanobody could not have a multivalent function, as the ClearColi displaying TY1 failed to form cross‐links in the agglutination assay. Therefore, we selected ClearColi displaying NB6 and VE as the candidates for further investigation.

### 2.3 SimCell Mediated Protein–Protein Blocking Assay to SARS‐COV‐2 RBD

**FIGURE 3:**
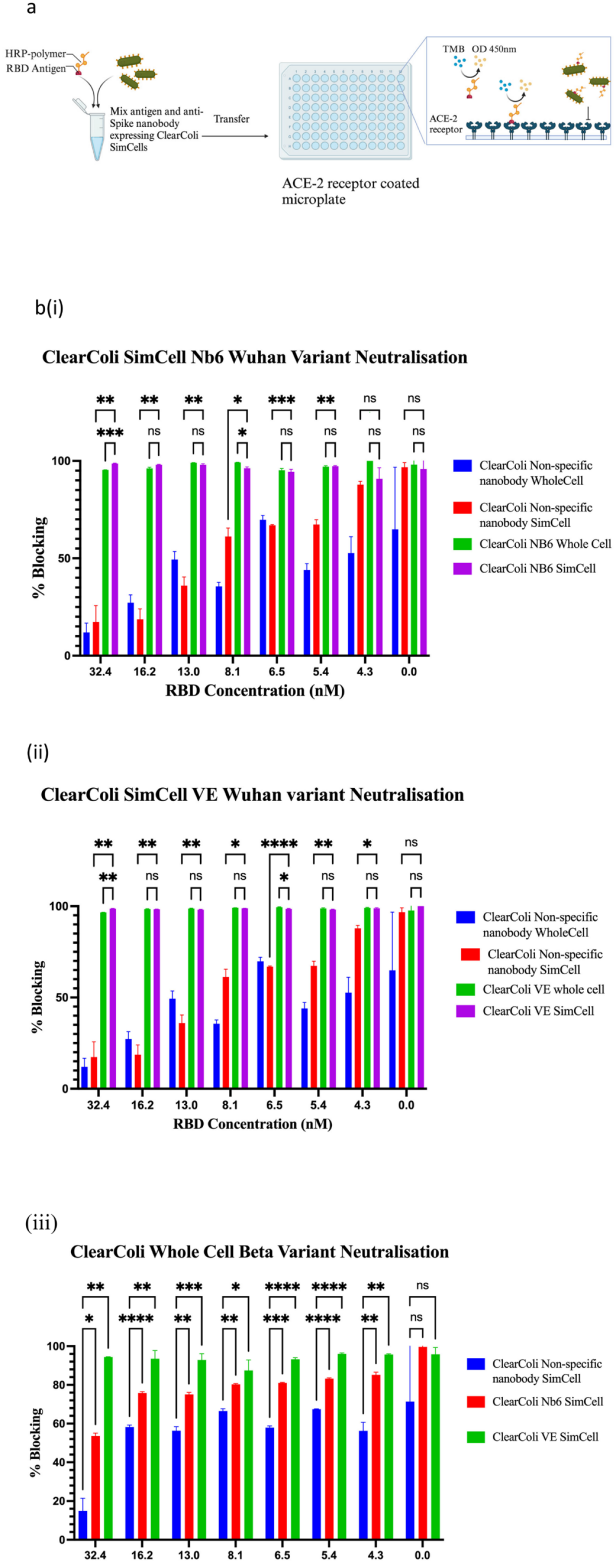
SimCell protein–protein Sars‐Cov‐2 RBD competitive ELISA (a) A schematic of the workflow for the RBD‐hACE2 competitive ELISA. Purified nanobody‐displaying SimCells were induced, purified, and diluted to OD600 = 2 with 1× PBS. The diluted SimCells were pre‐incubated with HRP‐conjugated RBD for 1 h statically at 37°C. The pre‐incubation mixture was then added to ACE2‐coated 96‐well microplates. Colorimetric measurement was performed using the chromogenic substrate 3,3′,5,5′‐tetramethylbenzidine (TMB) (Invitrogen), which reacts with HRP on the RBD. Subsequently, the stop solution was added to yield a yellow colour, measurable at 450 nm using a Tecan Spark plate reader. A high OD450nm reading indicates significant binding of HRP‐RBD to ACE2 on the plate, whilst a low or no OD450nm reading suggests minimal or no binding of HRP‐RBD to ACE2. (b) Neutralisation Assays with Wuhan variant RBD: (i) using pNV_Nb6 sfGFP whole‐cell and pNV_Nb6 sfGFP SimCell and (ii) pNV_VE sfGFP whole‐cell and pNV_VE sfGFP SimCell; neutralisation was compared with controls showing no binding and non‐specific counterparts anti‐HER2, labelled as unspecific cells. Wuhan HRP‐RBD concentrations of 0, 4.3, 5.4, 6.5, 8.1, 13, 16.2 and 32.4 nM were used. Both nanobody‐displaying whole cells and purified SimCells were washed and diluted with 1× PBS, then HRP‐RBD pre‐incubated with the washed cells for 1 h at 37°C before addition to the ACE2‐coated plate for 1 h at room temperature. The microplate was washed five times with 1× PBST to remove unbound HRP‐RBD, followed by the sequential addition of an equal volume of TMB and stop solution to yield an OD450nm reading, indicative of RBD‐hACE2 binding. Error bars represent the standard deviation from three biological replicates (*n* = 3). (iii) Competitive ELISA with the South African (Beta) variant RBD using pNV_Nb6 sfGFP whole‐cell and bispecific pNV_VE sfGFP whole‐cell; blocking efficiency was compared with controls showing no binding and non‐specific counterparts anti‐HER2, identified as the unspecific cell. South African variant HRP‐RBD concentrations of 0, 4.3, 5.4, 6.5, 8.1, 13, 16.2 and 32.4 nM were selected. Error bars represent the standard deviation from three biological replicates (*n* = 3). *p ≤ 0.05; **p ≤ 0.01; ***p ≤ 0.001; ****p ≤ 0.0001.

To investigate blocking efficacy, two hypotheses involving NB6 and VE were focused on. NB6 is known for its highly sensitive binding to the RBD at low concentrations, whilst VE can bind to two distinct RBD epitopes, potentially mitigating viral mutational escape. An in vitro competitive ELISA assay was utilised to replicate the hACE2‐RBD protein–protein interaction, mimicking the hACE2 receptor–virus interaction (Figure 3a). This assay tested the blocking efficiency of pNV_NB6_sfGFP and pNV_VE_sfGFP SimCells against the Wuhan SARS‐CoV‐2 variant RBD and the South African (Beta) SARS‐CoV‐2 variant RBD, both sourced from GenScript, in their binding to hACE2 provided by Thermo Fisher (Figure 3b). The negative control for the assay was pNV_anti‐HER2, a nanobody targeting HER2, which lacks specificity for the SARS‐CoV‐2 RBD. After washing with 1× PBST to remove any unbound HRP‐RBD, a significant decrease in the colorimetric signal was observed with NB6‐expressing ClearColi and its SimCells, indicating a > 95% blockade of the RBD‐hACE2 interaction (Table 1 and Figure S3a).

**TABLE 1:** The average blocking efficiency of pNV_Nb6 sfGFP whole‐cell, pNV_Nb6 sfGFP SimCell, pNV_VE sfGFP whole‐cell and pNV_VE sfGFP SimCell for blocking Wuhan and South African variant RBDs.

	Wuhan RBD	Beta RBD
NB6 whole cell	95.4% ± 0.07%	
NB6 SimCell	98.7% ± 0.18%	53.7% ± 1.39%
VE whole cell	96.6% ± 0.07%	
VE SimCell	98.6% ± 0.16%	94.4% ± 0.10%

*Note:* The average blocking efficiency at the highest RBD concentration (32.4 nM). Efficiency is calculated using a calibrated standard curve shown in Figure S3b.

### 2.4 SimCells and Mini‐SimCells Neutralisation of Live Virus

**FIGURE 4:**
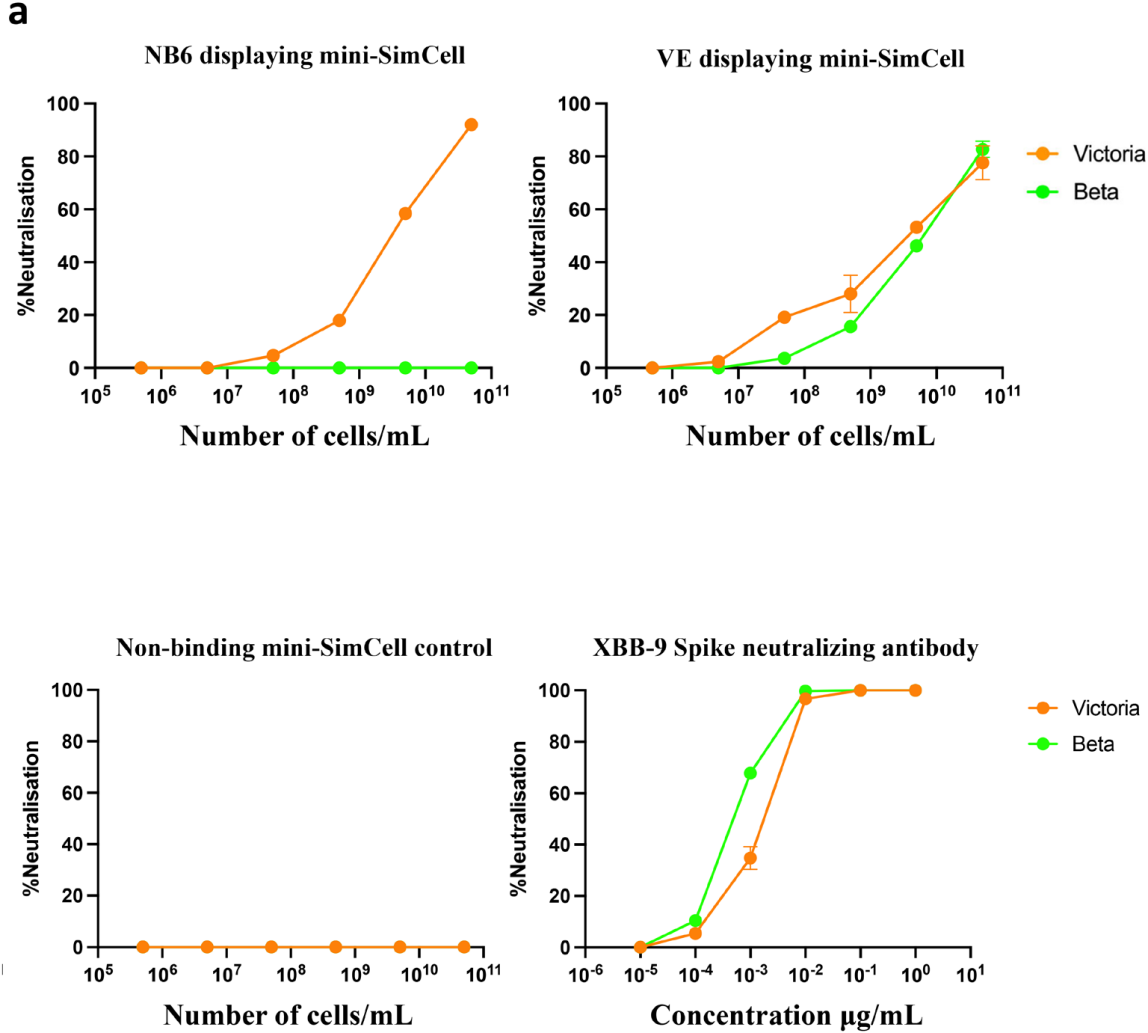
Neutralisation curves of anti‐S RBD nanobody displaying mini‐SimCell. (a) Neutralisation curves of NB6 anti‐Spike RBD monomeric nanobody‐expressing ClearColi mini‐SimCells and VE anti‐Spike RBD bivalent nanobody‐expressing ClearColi mini‐SimCells against the Victoria and B.1.351 (Beta) variants. Mini‐SimCells expressing a non‐binding (anti‐HER2) nanobody served as a negative control. Mini‐SimCells were serially diluted 10‐fold dilution for five times, starting from 5 × 10^10^/mL cells down to 5 × 10^5^/mL cells. For the assay, 50 μL of the mini‐SimCell samples were mixed with 200 foci/25 μL/well of viral particles. The XBB‐9 antibody, known for its neutralising capability against both Victoria and Beta variants, was used as a positive control. Error bars represent the average from two plate replicates, each contains two technical replicates (*n* = 4).

It was demonstrated that NB6 and VE displaying SimCells effectively bind the Spike RBD in a protein–protein in vitro competitive ELISA assay (Figures 1, 2, 3). Further evaluation of the neutralisation efficacy of the mini‐SimCells against live SARS‐CoV‐2 variants was conducted. The neutralisation assays were performed using NB6 and VE expressing mini‐SimCells against the Victoria strain (SARS‐CoV‐2/human/AUS/VIC01/2020) and the South African B.1.351 variant (Figure 4a). The live virus neutralisation assay performed with SARS‐CoV‐2 Victoria on Vero E6 cells infection, demonstrated neutralising activity for both NB6 and VE displaying mini‐SimCells with IC50 values of 2.95 × 10^9^ ± 1.40 × 10^8^ and 3.60 × 10^9^ ± 1.53 × 10^9^ cells/mL, respectively (Table 2). When assessing the neutralisation against the South African B.1.351 variant using the live virus neutralisation assay, VE displaying mini‐SimCells yielded an IC50 of 5.68 × 10^9^ ± 9.94 × 10^8^ cells/mL, whilst NB6 displaying mini‐SimCells showed no neutralisation effect (Table 2). These findings are consistent with earlier findings from protein–protein competitive ELISA assays, underscoring the specific neutralising capability of VE‐displaying mini‐SimCells against different variants. In addition, the neutralisation effect of NB6 SimCells was evaluated, yielding an IC50 of 4.78 × 10^8^ cells/mL, which is shown in Figure S4 and Table S3.

**TABLE 2:** The half‐maximal inhibitory concentration (IC50) of each experimental group against the Victoria and Beta viral variants. IC50 (number of cells/mL).

	Victoria	Beta
NB6	2.95 × 10^9^ ± 1.40 × 10^8^	> 5 × 10^10^
VE	3.60 × 10^9^ ± 1.53 × 10^9^	5.68 × 10^9^ ± 9.94 × 10^8^
Non‐binding control	> 5 × 10^10^	> 5 × 10^10^

## 3 Discussion

The economic and social repercussions of the COVID‐19 pandemic continue to exert a profound impact on global society. The emergence of highly transmissible and potentially vaccine‐resistant variants highlights the pressing need for cost‐effective prevention and treatment strategies to complement vaccines in addressing future viral threats. Neutralising nanobodies have emerged as promising candidates, demonstrating significant preclinical efficacy (Cox et al. 2022). Additionally, recent studies by Brüssow (2023, 2024) have emphasised the potential of vesicle‐based nasal vaccines to stimulate robust mucosal immunity, offering an innovative and complementary approach to systemic immunisation strategies. These vaccines provide a versatile solution to combat viral variants that may compromise vaccine effectiveness, presenting a vital component of an integrated response to emerging infectious diseases.

In response to this challenge, our study explored the use of a modular nanobody display system in LPS‐free ClearColi. We demonstrated the system's flexibility by readily expressing four distinct SARS‐CoV‐2 nanobodies (TY1, NIH‐CoVnb‐112, Nb6 and the bivalent nanobodies VE) in a ‘plug‐and‐play’ fashion. Our simple cell agglutination assay confirmed the engineered bacteria's ability to bind the desired antigen, SARS‐CoV‐2's RBD, with pNV_Nb6 sfGFP detecting antigen concentrations as low as 0.83 nM (Figure 2b). Notably, the multivalent nanobody VE showed binding capabilities up to 4.2 nM of RBD (Figure 2b), showing the potential for broad‐spectrum neutralisation.

To simulate virus‐host interactions, we developed an in vitro protein–protein blocking assay using human ACE2 and HRP‐conjugated RBDs. The nanobody‐displaying SimCells showed highly effective blocking capabilities, with NB6 and VE displaying SimCells inhibiting the interactions between the SARS‐CoV‐2 RBD and hACE2 by 98.7% and 98.6%, respectively (Table 1). Furthermore, the bivalent nanobody VE achieved more than 94.4% neutralisation against the RBD of the South African variant B.1.351. To evaluate the neutralisation efficiency of our nanosized mini‐SimCells, we conducted a *live* virus neutralisation assay. The IC50 values of NB6 and VE expressing mini‐SimCells were determined to be 2.95 × 10^9^ ± 1.40 × 10^8^ and 3.60 × 10^9^ ± 1.53 × 10^9^ mini‐SimCells/mL against the Victoria variant, respectively. VE also demonstrated a neutralisation effect against the Beta variant with an IC50 of 5.68 × 10^9^ ± 9.94 × 10^8^ mini‐SimCells/mL. Given that the molecular weight of XBB‐9 monoclonal antibody is 150 kDa, its IC50 is ~4 × 10^9^ XBB‐9/mL (Figure 4 and Table 2), indicating a comparable neutralisation effect. The production of mini‐SimCells eliminates the need for monoclonal antibody purification, and minicell safety have been confirmed in human clinical trials (van Zandwijk et al. 2017; Ganju et al. 2024). These results highlight the modularity and potential of nanobody‐displaying SimCells and mini‐SimCells as powerful tools for neutralising SARS‐CoV‐2 and its emerging variants.

This technology demonstrates significant potential for application in humans as part of future strategies to protect against viral infections. However, further investigations are necessary to ensure its feasibility and safety. The application of this approach requires a structured development pathway, including preclinical studies to evaluate safety, pharmacokinetics, biodistribution and immunogenicity in animal models. Subsequently, clinical trials should be conducted in phases, beginning with Phase I trials to assess safety in healthy volunteers, followed by Phase II and III trials to evaluate efficacy, optimal dosing and long‐term safety in diverse populations.

In conclusion, this study underscores the potential of nanobody‐displaying SimCells as an innovative therapeutic platform for SARS‐CoV‐2 and its variants. These systems represent a cost‐effective and scalable approach, complementing existing vaccine strategies and contributing to a comprehensive framework for pandemic preparedness and response. Their integration into broader public health strategies has the potential to enhance global resilience against current and future viral threats.

## 4 Experimental Procedures

### 4.1 Strains, Cell Cultures and DNA Manipulation

In this study, we utilised competent *E. coli* DH5α strains for routine cloning and plasmid maintenance. For the expression of nanobodies and subsequent experiments, *E. coli* ClearColi (DE3) strains were employed, unless specified otherwise.

All *E. coli* cells were cultured in LB Miller broth (Sigma Aldrich) and plated on LB Miller agar (Sigma Aldrich), followed by incubation at 37°C with shaking at 250 rpm to ensure proper aeration. For SimCell conversion, we utilised M9 minimal medium. The 5× M9 minimal salts (Sigma Aldrich) composition included KH2PO4 (15 g/L), NaCl (2.5 g/L), Na2HPO4 (33.9 g/L) and NH4Cl (5 g/L). The 1× M9 medium was supplemented with 1× trace elements (prepared according to the recipe available at: https://static.igem.org/mediawiki/2019/2/20/T‐Tuebingen‐M9_recipe.pdf), 1 mM MgSO_4_, 0.3 mM CaCl_2_, 0.4% (w/v) glucose and 0.2% (w/v) casamino acids. The antibiotics used were carbenicillin at 100 μg/mL, chloramphenicol at 34 μg/mL and kanamycin at 50 μg/mL.

All enzymes required for molecular cloning were sourced from New England Biolabs (NEB). Plasmid construction was carried out using NEBuilder HiFi DNA Assembly, and DNA amplification for assembly purposes was conducted with Q5 high‐fidelity DNA polymerase.

### 4.2 *Escherichia coli* Competent Cell Preparation and Transformations

General molecular cloning was performed using NEB 5‐α high‐efficiency competent cells. To prepare competent *E. coli* cells (DH5α/ClearColi BL21(DE3)), we initiated the process by growing an overnight culture in an LB medium. The following day, we diluted 1 mL of the overnight culture into 50 mL of LB broth in a sterile 250 mL Erlenmeyer flask and incubated the culture at 37°C with shaking at 150 rpm until the OD600 reached 0.5–0.6. During this growth period, we prepared and filter‐sterilised 25 mL each of 0.1 M CaCl_2_ and 0.1 M MgCl_2_ solutions, keeping them on ice. After the desired OD600 was achieved, the culture was cooled on ice for 30 min, then transferred to a 50 mL Falcon tube and centrifuged at 1000 *g* for 10 min at 4°C. The supernatant was discarded, and the cell pellet was gently resuspended in chilled 25 mL of 0.1 M MgCl_2_. This centrifugation step was repeated, and the pellet was resuspended in chilled 25 mL of 0.1 M CaCl_2_. Following the final centrifugation, the supernatant was discarded and the cells were resuspended in 900 μL of CaCl_2_. We then added 600 μL of CaCl_2_ and 400 μL of 50% glycerol solution, using 50% w/v sterile glycerol to achieve the correct concentration. The final suspension of competent cells was now ready for transformations or storage for later use.

For heat‐shock transformation, we combined 50 μL of chemically competent cells with either 100 ng of plasmid DNA or 5 μL of a HiFi assembly product, mixing by flicking. The transformation protocol was as follows: incubation at 4°C on ice for 30 min, a 42°C heat shock in a water bath for 45 s, followed by a 2‐min rest on ice. Then, we added 950 μL of LB or SOC media to the cells and allowed them to recover at 37°C for 1 h. Post‐recovery, the transformed cells were centrifuged at 3000 *g* for 5 min, resuspended in 100 μL of LB and plated onto LB agar plates with the appropriate antibiotics.

For strains carrying two plasmids, chemically competent cells harbouring the first plasmid were prepared and used for sequential heat‐shock transformation with the second plasmid.

In cases of difficult transformation with ClearColi BL21(DE3), we utilised electrocompetent cells purchased from LCG Biosearch Technologies and followed a standard electroporation protocol.

### 4.3 Immunostaining and Flow Cytometry

Flow cytometry was used to verify the pNV nanobody presentation on the surface of bacterial strains. Overnight bacterial culture carrying nanobody expression system was centrifuged at 4°C, 2000 *g* for 5 min. A 1 mL non‐fat milk‐blocking buffer (1%) in phosphate‐buffered saline (PBS) was added to resuspend the cell pellet and incubated at room temperature for 1 h. Blocked culture was transferred to a 1.5 mL Eppendorf tube and was centrifuged at 4000 *g* for 2 min. The pellet was washed twice and resuspended in 1 mL of PBS. A 2 μL of primary anti‐Myc antibody (Abcam, ab9106) was added to the 1 mL cell suspension and incubated at room temperature for 1.5 h. The suspension was centrifuged at 1000 *g* for 5 min and washed twice with 1 mL of PBS. The pellet was resuspended in 500 μL of PBS and added with 0.5 μL of secondary Alexa Fluor 488 conjugated antibody (Abcam, ab150077). The culture was incubated at room temperature for 1 h. Finally, the culture was centrifuged at 1000 *g* for 5 min, washed twice and resuspended in 1 mL of PBS.

Cell fluorescence was measured using an FACS Calibur (BD Biosciences) with FL1 filter to detect Alexa Fluor 488 has an excitation/emission at wavelength 488/530 nm. Fluorescence data was collected from 100,000 viable cells for each experiment using CellQuest and analysed using FlowJo software.

### 4.4 Cell Agglutination Assay

Overnight cultures of pNV_sfGFP nanobody‐displaying strains were centrifuged at 2000 *g* at 4°C for 5 min and washed three times with 1× PBS, followed by dilution in 1× PBS to an OD600 of 0.8. 100 μL of this diluted culture was then added to a U‐shaped, round‐bottom 96‐well plate containing varying concentrations of SARS‐CoV‐2 RBD (Thermo Fisher). The plate was incubated statically overnight at room temperature. Subsequently, a top‐view image was captured using the VersaDoc Imaging System (Bio‐Rad) under the FITC channel.

### 4.5 Cell Adhesion Assay

ELISA plates (Nunc, MaxiSorp) were coated with 100 μL per well of RBD antigen diluted in PBS (pH 7.4) to the desired concentration and incubated overnight at 4°C. The plates were washed three times with a washing buffer consisting of 0.05% Tween‐20 in PBS. Overnight pNV_sfGFP cultures were centrifuged at 2000 *g* at 4°C for 5 min and washed three times with 1× PBS. The resulting cell pellet was resuspended in 600 μL of 1% skimmed milk in PBST and incubated at room temperature for 1 h. The blocked cultures were then centrifuged and washed with 1× PBS, followed by dilution in 1× PBS to an OD600 of ~1. We added 100 μL of the diluted cultures into individual wells and incubated them at room temperature for 1 h. After incubation, the cell cultures were removed, and the wells were washed four times with PBST. Finally, a fluorescence image was taken using a BioTek imager.

### 4.6 Batch Production of Pure SimCell

Overnight cultures of ClearColi (DE3) harbouring the endonuclease ICeuI were prepared in 5 mL volumes and subsequently diluted 1:100 in 50 mL of M9 minimal media, supplemented with 0.4% glucose and 0.2% casamino acids, in an Erlenmeyer flask. This flask was incubated in a shaking incubator at 37°C and 250 rpm overnight. Following this incubation, 50 mL of the culture was centrifuged at 1000 *g* for 10 min and washed three times with minimal media, also supplemented with 0.2% casamino acids. The cell pellet was then resuspended in minimal media containing 0.2% casamino acids and 1 μM Crystal Violet, serving as the inducer for ICeuI expression to initiate SimCell conversion. This resuspension was transferred to an Erlenmeyer flask and incubated at 37°C, 250 rpm overnight before the addition of antibiotics: cefotaxime (100 μg/mL) and penicillin G (100 μg/mL) to eradicate dividing parental cells. The purified SimCell culture underwent further incubation at 37°C and 250 rpm for 24 h. Our findings indicate that the ClearColi SimCells generated using this method result in uniformly sized SimCells, optimising therapeutic outcomes and reducing the occurrence of filamentous chromosome‐less SimCells. The resultant genome‐less SimCells can be stored at 4°C for later use or, for immediate application, centrifuged at 1000 *g* for 10 min at 4°C and washed three times with 1× PBS. The PBS‐washed culture is then ready for immediate use or can be stored at 4°C for later use. Detailed protocol and characterisation are shown in Figure S2.

To assess the purity of the SimCell, we plated 2 μL of both uninduced ClearColi 12× and induced ClearColi SimCell cultures on LB agar plates for CFU counting. No colonies were observed for the induced SimCell culture after 72 h (Figure S1b).

### 4.7 Characterisation of SimCells Conversion

ClearColi (DE3) was transformed with the ICeuI plasmid, referred to as 12×. Glycerol stocks of strains containing the 12× plasmid were streaked onto LB‐agar plates containing antibiotics and incubated overnight at 37°C. Single colonies were selected from these plates and inoculated into 5 mL of LB medium supplemented with the appropriate antibiotic, then incubated overnight at 37°C at 250 rpm. A 2 μL of these overnight cultures were reinoculated into an M9 medium supplemented with 0.4% glucose and 0.2% casamino acids, and incubated at 37°C, 250 rpm. A 2 μL of the overnight culture was then diluted in 198 μL of M9 medium supplemented with 0.4% glucose, 0.2% casamino acids and the corresponding antibiotics. These cultures were transferred to the wells of a 96‐well microplate (Costar), which was sealed with a Breathe‐Easy sealing membrane. The plate was placed in a Synergy2 microplate reader (BioTek) and incubated at 37°C with orbital shaking at 100 rpm for 5 h. The 96‐well plate was subsequently removed, and 1 μM of Crystal Violet, along with penicillin G (100 μg/mL), was added to each well. The plate was then returned to the incubator for an additional 40 h (Figure S1a).

### 4.8 Production and Purification of Mini‐SimCell

Overnight cultures containing plasmids for the ClearColi ΔminD strain were initiated from 5 mL samples, which were then diluted 1:100 into 50 mL of LB medium in an Erlenmeyer flask. This setup was incubated at 37°C with shaking at 250 rpm, reaching an optical density at 600 nm (OD600) between 2 and 5 overnight.

Following incubation, the culture was transferred into a 50 mL Falcon tube and centrifuged at 2000 *g* for 10 min at 4°C, preserving the supernatant for a subsequent centrifugation step at 12,000 *g* for 15 min at the same temperature. The pellet obtained was resuspended in 5 mL of fresh LB medium supplemented with ceftriaxone (100 μg/mL), penicillin G (100 μg/mL) and cefotaxime (100 μg/mL), and the mixture was incubated again at 37°C with shaking at 250 rpm for a period ranging from 4 h to overnight.

To eliminate cell debris, the culture was first centrifuged at 200 *g* for 10 min at 4°C, and the clear supernatant was collected for final centrifugation at 16,000 *g* for 15 min at 4°C. The quantity of mini‐SimCells was determined by measuring the OD600 with a NanoVue Plus Spectrophotometer (GE Healthcare), employing the following formula for precise quantification (Giacalone et al. 2006; Jivrajani et al. 2012)

$$N_{\text{minicells}}=A_{600}\times 5.0\times 10^{10}/\mathrm{mL}$$

The collected pellet was then redissolved in 1 mL of PBS and kept at 4°C for subsequent use. This refined preparation is identified as mini‐SimCells.

### 4.9 RBD Protein–Protein In Vitro Blocking Assay

For this study, a MaxiSORP ELISA plate (Nunc) was coated with 100 ng of human ACE‐2 (hACE‐2) suspended in 50 μL of 100 mM carbonate–bicarbonate coating buffer (pH 9.6) and incubated at 4°C overnight. After incubation, the plate was washed four times with 1× PBST and blocked with SuperBlock (Thermo Fisher) blocking buffer. Once the blocking buffer had dried completely, the plate was ready for immediate use or could be stored at 4°C for future experiments.

HRP‐conjugated SARS‐CoV‐2 (GenScript) was quantified using the Thermo Fisher QuanT‐iT protein quantification kit. In the no‐binding assay, a dilution series of HRP‐conjugated SARS‐CoV‐2 Wuhan and South African B.1.351 (Beta) variant RBDs (produced by GenScript) ranged from 4.3 to 32.4 nM. These dilutions were added to the hACE‐2‐coated plates in 50 μL of PBS for 1 h at room temperature. Unbound HRP‐conjugated RBD was then washed away with phosphate‐buffered saline containing 0.05% Tween‐20 (PBST) five times. For colourimetric measurement, 100 μL of TMB chromogenic substrate (Invitrogen) was added and incubated for 15 min to allow the enzymatic reaction with HRP to occur. The reaction was stopped using an equal volume of TMB stop solution, and absorbance at 450 nm was read using a Tecan Spark plate reader.

For the whole cell or SimCell nanobody‐expressing blocking assay, the same SARS‐CoV‐2 HRP‐RBD dilution series were pre‐incubated with 50 μL of PBS‐diluted bacterial whole cells/SimCells at OD = 2 for 1 h at 37°C. This mixture was then added to a MaxiSORP ELISA plate coated with hACE‐2 (100 ng per well) and incubated for 1 h at room temperature. After five PBST washes to remove unbound antigens, the inhibition efficiency was calculated using standard curves (see Figure S3b).

### 4.10 Live Virus Neutralisation Assay

SimCells and mini‐SimCell were serially diluted and mixed with SARS‐CoV‐2 strains, followed by incubation for 1 h at 37°C. XBB‐9 antibody was used as a positive control for the neutralisation of SARS‐CoV‐2. Subsequently, these mixtures were transferred to 96‐well, cell culture‐treated, flat‐bottom microplates that contained confluent Vero cell monolayers, duplicated and incubated for an additional 2 h. Afterwards, 1.5% semi‐solid carboxymethyl cellulose (CMC) overlay medium was added to each well to restrict virus diffusion; 20–24 h later, a focus forming assay was conducted. This involved fixing the Vero cells with 4% formaldehyde, permeabilizing with 2% Triton X‐100 in PBS and then staining the Vero cells with an in‐house mouse anti‐NP monoclonal antibody (Dejnirattisai et al. 2021), followed by a peroxidase‐conjugated goat anti‐mouse IgG (Sigma, A0170). The foci (infected cells), which number ~200 per well in the absence of mini‐SimCell, were visualised by the addition of TrueBlue Peroxidase Substrate. The virus‐infected cell foci were counted using the classic AID EliSpot reader and AID ELISpot software. The percentage of focus reduction was calculated, and the IC50 was determined using the probit program from the SPSS package. This experiment was done twice as separate plate assays, and each plate contains two biological replicates. Therefore, standard error (SEM) was calculated for statistical analysis.

## Author Contributions

**Yutong Yin:** investigation, writing – original draft, data curation, writing – review and editing, methodology, formal analysis. **Chang Liu:** investigation, resources, writing – review and editing, validation. **Xianglin Ji:** writing – review and editing, resources. **Yun Wang:** writing – review and editing, resources. **Juthathip Mongkolsapaya:** writing – review and editing, supervision, resources, validation. **Gavin R. Screaton:** supervision, resources, writing – review and editing, validation. **Zhanfeng Cui:** supervision, resources, writing – review and editing. **Wei E. Huang:** conceptualization, methodology, supervision, resources, writing – review and editing, funding acquisition.

## Conflicts of Interest

Y.Y., X.J., H.W., Z.C. and W.E.H. have filed a provisional patent application with the UK Patent Office related to this work.

## Supporting information


Data S1.

## Data Availability

The data that supports the findings of this study are available in the Supporting Information of this article.
