# Supplementary material for: Engineering Genome‐Free Bacterial Cells for Effective SARS‐COV‐2 Neutralisation
Source: Microb Biotechnol. 2025 Mar 5;18(3):e70109. doi: 10.1111/1751-7915.70109 (PMC11881285; doi:10.1111/1751-7915.70109)
Supplement: Supplementary file 1 — Data S1. [file MBT2-18-e70109-s001.pdf]

Supplementary Information

Engineering genome-free bacterial cells for effective SARS-COV-2 neutralisation

Yutong Yin[1], Chang Liu[2,3], Xianglin Ji[4], Yun Wang[4], Juthathip Mongkolsapaya[2,3,5] , Gavin R. Screaton[2,3], Zhanfeng Cui[1,6], Wei E. Huang[1]*.

1. Department of Engineering Science, University of Oxford, Parks Road, OX1 3PJ, Oxford, UK.
2. Wellcome Centre for Human Genetics, Nuffield Department of Medicine, University of Oxford, Oxford OX3 7BN, UK.
3. Chinese Academy of Medical Science (CAMS) Oxford Institute (COI), University of Oxford, Oxford OX3 7FZ, UK.
4. Oxford Suzhou Centre for Advanced Research (OSCAR), University of Oxford, Suzhou Industrial Park, Jiangsu, China
5. Mahidol-Oxford Tropical Medicine Research Unit, Bangkok, Thailand, Department of Medicine, University of Oxford, Oxford, UK
6. Institute of Biomedical Engineering, Department of Engineering Science, University of Oxford, OX3 7DQ, UK.

*Corresponding author
Wei E. Huang
Tel.: +44 1865 283786
Email: wei.huang@eng.ox.ac.uk

Keywords: synthetic biology, SimCell, mini-SimCell, bacterial therapy, chromosome-free, synthetic cell, live virus neutralisation

Table S1. Bacterial strains and plasmids in this study.

**Strains**	**Genotype description**	**Source**
Escherichia coli DH5α	*fhuA2 lac(del)U169 phoA glnV44 Φ80' lacZ(del)M15 gyrA96 recA1 relA1 endA1 thi-1 hsdR17*	Lab collection
Escherichia coli ClearColi BL21(DE3)	*F– ompT hsdSB (rB- mB-) gal dcm lon λ(DE3 [lacI lacUV5-T7 gene 1 ind1 sam7 nin5]) msbA148 ΔgutQΔkdsD ΔlpxLΔlpxMΔpagPΔlpxPΔeptA*	Purchased from Lucigen[1]
ClearColi *ΔminD*	*F– ompT hsdSB (rB- mB-) gal dcm lon λ(DE3 [lacI lacUV5-T7 gene 1 ind1 sam7 nin5]) msbA148 ΔgutQΔkdsD ΔlpxLΔlpxMΔpagPΔlpxPΔeptA ΔminD*	This study
ClearColi Negative control (pNV_J23100)	ClearColi: for FACS negative control (high expression of nanobody displaying system without VHH)	This study
ClearColi Negative control (pNV_J23100)	ClearColi: for FACS negative control (high expression of nanobody displaying system without VHH)	This study
ClearColi pNV_J23100_TY1 VHH_	ClearColi high expression of TY1 VHH[2] display	This study
ClearColi pNV_J23100_NB6 VHH	ClearColi high expression of NB6 VHH[3] display	This study
ClearColi pNV_J23100_NIH112 VHH	ClearColi high expression of NIH112 VHH[4] display	This study
ClearColi pNV_J23100_VE VHH	ClearColi high expression of VE VHH [3]display	This study
ClearColi pNV_J23100_NB6 VHH_sfGFP + pRH12X(I-CeuI)	ClearColi: for SimCell production (high expression of VHH display with sfGFP downstream)	This study
ClearColi *ΔminD* pNV_J23100_NB6 VHH_sfGFP	ClearColi: for mini-SimCell production (high expression of NB6 VHH display with sfGFP downstream)	This study
ClearColi *ΔminD* pNV_J23100_VE VHH_sfGFP	ClearColi: for mini-SimCell production (high expression of VE VHH display with sfGFP downstream)	This study
**Plasmids**		
Negative control (pNV_J23100)	Plasmid with constitutively promoter BBa_J23100 and an RBS 12K, expressing β-intimin as the outer. CmR.	This study
pNV_J23100 non-binding VHH	Plasmid with constitutively promoter BBa_J23100 and an RBS 12K, expressing β-intimin as the outer membrane with anti-HER2 (VHH) fused to it. CmR.	This study
pNV_J23100 TY1 VHH	Plasmid with constitutively promoter BBa_J23100 and an RBS 12K, expressing β-intimin as the outer membrane with anti-spike RBD nanobody TY1(VHH) fused to it. CmR.	This study

pNV_J23100 NIH112 VHH	Plasmid with constitutively promoter BBa_J23100 and an RBS 12K, expressing β-intimin as the outer membrane with anti-spike RBD nanobody NIH112(VHH) fused to it. CmR.	This study
pNV_J23100_NB6 VHH	Plasmid with constitutively promoter BBa_J23100 and an RBS 12K, expressing β-intimin as the outer membrane with anti-spike RBD nanobody NB6(VHH) fused to it. CmR.	This study
pNV_J23100_VE VHH	Plasmid with constitutively promoter BBa_J23100 and an RBS 12K, expressing β-intimin as the outer membrane with anti-spike RBD bivalent nanobody VE(VHH) fused to it. CmR.	This study
pNV_J23100 non-binding VHH_sfGFP	Plasmid with constitutively promoter BBa_J23100 and an RBS 12K, expressing β-intimin as the outer membrane with anti-HER2 (VHH) fused to it and sfGFP controlled by RBS BBa_B0034. CmR.	This study
pNV_J23100_TY1 VHH_sfGFP	Plasmid with constitutively promoter BBa_J23100 and an RBS 12K, expressing β-intimin as the outer membrane with anti-spike RBD TY1 (VHH) fused to it and sfGFP controlled by RBS BBa_B0034. CmR.	This study
pNV_J23100_NIH112 VHH_sfGFP	Plasmid with constitutively promoter BBa_J23100 and an RBS 12K, expressing β-intimin as the outer membrane with anti-spike RBD NIH112 (VHH) fused to it and sfGFP controlled by RBS BBa_B0034. CmR.	This study
pNV_J23100_NB6 VHH_sfGFP	Plasmid with constitutively promoter BBa_J23100 and an RBS 12K, expressing β-intimin as the outer membrane with anti-spike RBD NB6 (VHH) fused to it and sfGFP controlled by RBS BBa_B0034. CmR.	This study
pNV_J23100_VE VHH_sfGFP	Plasmid with constitutively promoter BBa_J23100 and an RBS 12K, expressing β-intimin as the outer membrane with anti-spike RBD VE (VHH) fused to it and sfGFP controlled by RBS BBa_B0034. CmR.	This study
pRH12x	Plasmid with ICeuI endonuclease controlled by a crystal violet inducible promoter. KanR.	This study

Table S2. Primers and DNA gblocks in this study.

Primers	Sequence (5’ -> 3’)	Purpose	Plasmids
Nanobody FWD	gtaaggcaaccgtaacgttgaagtcgagtac	Nanobody VHH sequence into the nanobody display system	pNV_J23100 TY1VHH_sfGFP; pNV_J23100 NIH112 VHH_sfGFP; pNV_J23100 NB6VHH_sfGFP; pNV_J23100 NB6VHH_sfGFP; pNV_J23100 non-binding VHH_sfGFP
Nanobody REV	tctcctctttTTATGCAGCTGCATCCTC		
Backbone FWD	agctgcataaAAAGAGGAGAAAGGTACCATG	pNV_J23100_sfGFP backbone	
Backbone FWD	caacgttacggttgccttaccgttagcatc		
**gblocks**			
Anti-Spike RBD VHH: TY1	gtaaggcaaccgtaacgttgaagtcgagtacgccaggacaggtcgtcgtgtctgctaaaaccgcggagatgacttcagcacttaatgccagtgcggttatatttttgatggtgcgccggtgccgtatccggatccgctggaaccggcccagccggccCAGGTGCAGCTCGTGGAGACGGGGGGAGGCTTGGTGCAGCCTGGGGGGTCTCTGAGACTCTCCTGTGCAGCCTCTGGATTCACCTTCAGTAGCGTCTACATGAACTGGGTCCGCCAGGCTCCAGGGAAGGGGCCCGAGTGGGTCTCGCGTATTAGTCCGAATAGTGGTAATATTGGGTATACAGACTCCGTGAAGGGCCGATTCACCATCTCCAGAGACAACGCCAAGAACACACTGTATCTGCAAATGAATAACCTGAAACCTGAGGACACGGCCCTGTATTACTGTGCGATTGGTTTGAATTTGAGTAGTAGCTCCGTTAGGGGCCAGGGGACCCAGGTCACCGTCTCCTCAgctcgagccgaacaaaaactcatctcagaaGAGGATGCAGCTGCATAAaaagaggaga	anti-spike RBD TY1 (VHH) (highlighted in yellow)	
Anti-Spike RBD VHH: NIH112	gtaaggcaaccgtaacgttgaagtcgagtacgccaggacaggtcgtcgtgtctgctaaaaccgcggagatgacttcagcacttaatgccagtgcggttatatttttgatggtgcgccggtgccgtatccggatccgctggaaccggcccagccggccGACGTACAGTTGCAGGAGAGTGGTGGCGGCCTGGTCCAGCCCGGCGGTTCTCTCCGGCTGTCATGTGCAGCGAGTGGTCTTACCTTAGATTACTACGCCATTGGCTGGTTTCGTCAAGCGCCTGGAAAAGAACGCGAAGGGGTGTCTTGCATCAGTTCGAGCGATGGAAGCACATACTATGCTGACTCCGTGAAAGGCCGCTTCACGACTAGCCGTGACAACGCTAAAAATACGGTTTATCTGCAGATGAACTCCCTGAAACCGGAAGATACAGCCGTCTATTACTGCGCGGCA	anti-spike RBD NIH112 (VHH) (highlighted in yellow)	

	GTTCCATCGACCTATTACAGCGGGA<br>CTTACTATTATACGTGTCATCCGGG<br>TGGGATGGATTATTGGGGCAAGGG<br>TACCCAAGTGACCGTATCGTCA	
Anti-Spike RBD VHH: NB6	gtaaggcaaccgtaacgttgaagtcgagtacgccaggac<br>aggtcgtcgtgtctgctaaaaccgcggagatgacttcagc<br>acttaatgccagtgcggttatattttttgatggtgcgccggt<br>gccgtatccggatccgctggaaccggcccagccggcc<br>CAGGTGCAACTTGTCGAATCGGGA<br>GGCGGCTTAGTTCAGGCGGGTGGA<br>TCTCTCCGCTTGTCCTGCGCAGCTA<br>GCGGTTATATCTTTGGGCGAAATGC<br>CATGGGTTGGTATCGTCAGGCTCCA<br>GGGAAGGAGCGCGAACTGGTCGCC<br>GGCATCACGCGGCGTGGCTCAATT<br>ACTTACTACGCGGACAGCGTAAAA<br>GGCCGTTTCACGATTAGTCGCGATA<br>ATGCAAAAAACACAGTATATCTGC<br>AAATGAACTCGCTGAAACCGGAAG<br>ATACCGCCGTGTATTACTGTGCAGC<br>GGACCCGGCGTCCCCTGCGTACGGT<br>GATTATTGGGGCCAGGGTACCCAG<br>GTTACCGTGAGCAGTCATCATCATC<br>ACCACCACgctcgagccgaacaaaaactcatctca<br>gaaGAGGATGCAGCTGCATAAaaagag<br>gaga	anti-spike RBD NB6 (VHH) (highlighted in yellow)
Anti-Spike RBD VHH: VE	gtaaggcaaccgtaacgttgaagtcgagtacgccaggac<br>aggtcgtcgtgtctgctaaaaccgcggagatgacttcagc<br>acttaatgccagtgcggttatattttttgatggtgcgccggt<br>gccgtatccggatccgctggaaccggcccagccggcc<br>CAGGTCCAGCTGGTTGAGACCGGG<br>GGCGGTCTTGTTCAGCCAGGCGGTA<br>GCCTGCGCCTGTCTTGCGCCGCGTC<br>AGGGTTTACCTTCTCAAGCTACGCA<br>ATGGGATGGGCCCGACAAGTACCA<br>GGTAAAGGTCTCGAATGGGTGTCCT<br>ATATTTATTCGGACGGCAGTACGGA<br>GTATCAAGATAGTGTGAAAGGTCG<br>CTTCACAATCAGCCGTGATAACGCT<br>AAAAGCACCGTGTACCTGCAAATG<br>AACTCCCTGAAACCGGAAGATACT<br>GCAGTTTATTATTGTGCTACCGAAG<br>GGAGTCTGGGAGGGTGGGGCCGGG<br>ATTTTGGTTCGTGGGGACAGGGCAC<br>ACAAGTAACGGTCTCTTCAGGCGG<br>CGGTGGCTCGGGTGGTGGGGGTTC<br>CGGGGGCGGCGGATCACAGGTCCA<br>GTTAGTCGAAACGGGCGGTGGTTTC<br>GTGCAACCCGGAGGCTCGCTGCGT<br>CTTTCTTGCGCGGCGTCGGGCGTTA<br>CGTTAGATTATTACGCTATTGGCTG<br>GTTTCGCCAGGCACCGGGCAAAGA<br>AAGAGAGGGCGTGTCTTGCATTGG<br>CTCCAGCGACGGCCGTACATACTAT<br>AGTGACAGCGTAAAGGGTCGTTTT	anti-spike bivalent RBD VE (VHH) (highlighted in yellow)

	ACCATCTCCCGCGACAATGCGAAA<br>AACACCGTTTACTTGCAGATGAATA<br>GCCTAAAGCCGGAAGACACCGCCG<br>TCTATTATTGTGCCTTGACCGTTGG<br>TACTTATTACTCTGGCAATTATCAC<br>TATACTTGTAGTGATGACATGGATT<br>ATTGGGGAAAAGGGACGCAGGTGA<br>CGGTGAGCAGCGGTGGGTACCCTT<br>ACGATGTGCCGGATTACGCGGGTC<br>ATCACCACCATCATCATgctcgagccgaa<br>caaaaactcatctcagaaGAGGATGCAGCTG<br>CATAAaaagaggaga	
Non-binding VHH (anti-Spike RBD)	gtaaggcaaccgtaacgttgaagtcgagtacgccaggac<br>aggtcgtcgtgtctgctaaaaccgcggagatgacttcagc<br>acttaatgccagtgcggttatattttttgatggtgcgccggt<br>gccgtatccggatccgctggaaccggcccagccggcc<br>CAGGTGCAGCTGCAGGAAAGCGGT<br>GGTGGTTCAGTGCAAGCAGGAGGC<br>AGCCTGAAACTCACCTGCGCGGCCT<br>CTGGCTACATTTTTAACTCGTGTGG<br>AATGGGGTGGTACCGCCAGAGCCC<br>GGGCCGCGAGCGTGAATTAGTCTC<br>GCGTATTTCTGGCGATGGCGATACG<br>TGGCATAAAGAAAGTGTCAAAGGT<br>CGGTTTACAATCTCACAGGATAACG<br>TAAAAAAGACTCTGTATCTTCAAAT<br>GAATAGTCTGAAACCAGAGGACAC<br>CGCTGTTTATTTCTGTGCGGTGTGC<br>TACAATTTGGAAACCTATTGGGGGC<br>AAGGTACTCAGGTAACGGTTTCCTC<br>CgctcgagccgaacaaaaactcatctcagaaGAGG<br>ATGCAGCTGCATAAaaagaggaga	Anti-HER2 nanobody (highlighted in yellow)

Table S3

This table presents the half-maximal inhibitory concentration (IC50) values for each experimental group against the Victoria viral variants.

IC50 (Number of Cells per mL)	
**NB6 SimCell**	4.78E+08
**NB6 mini-SimCell**	3.68E+09

Supplementary Figure S1

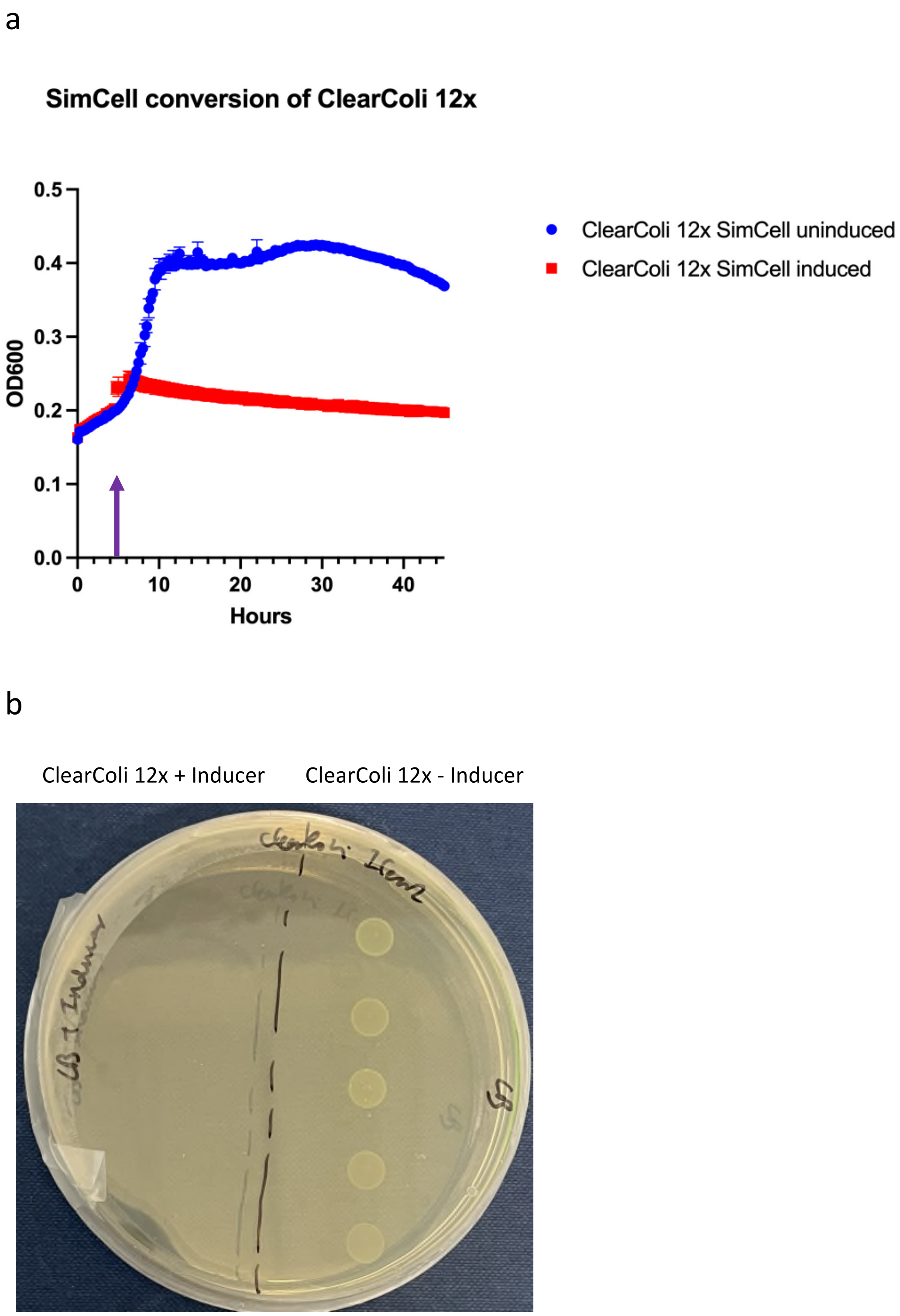

Supplementary Figure S1. (a) OD600 growth curve of the ClearColi strain containing ICeuI, referred to as ClearColi 12x, for SimCell conversion. The purple arrow marks the addition of

an inducer for ICeuI expression at the 3-hour time point. The blue line represents the growth curve of the uninduced ICeuI, while the red line depicts the growth curve following ICeuI induction and ClearColi SimCell conversion. Error bars indicate the standard deviation from three biological replicates. **(b)** 50 μL of either induced or uninduced ClearColi 12x culture from the growth curve experiment was spotted onto an LB-only agar plate and incubated overnight at 37°C in a static incubator to assess SimCell purity.

Supplementary Figure S2

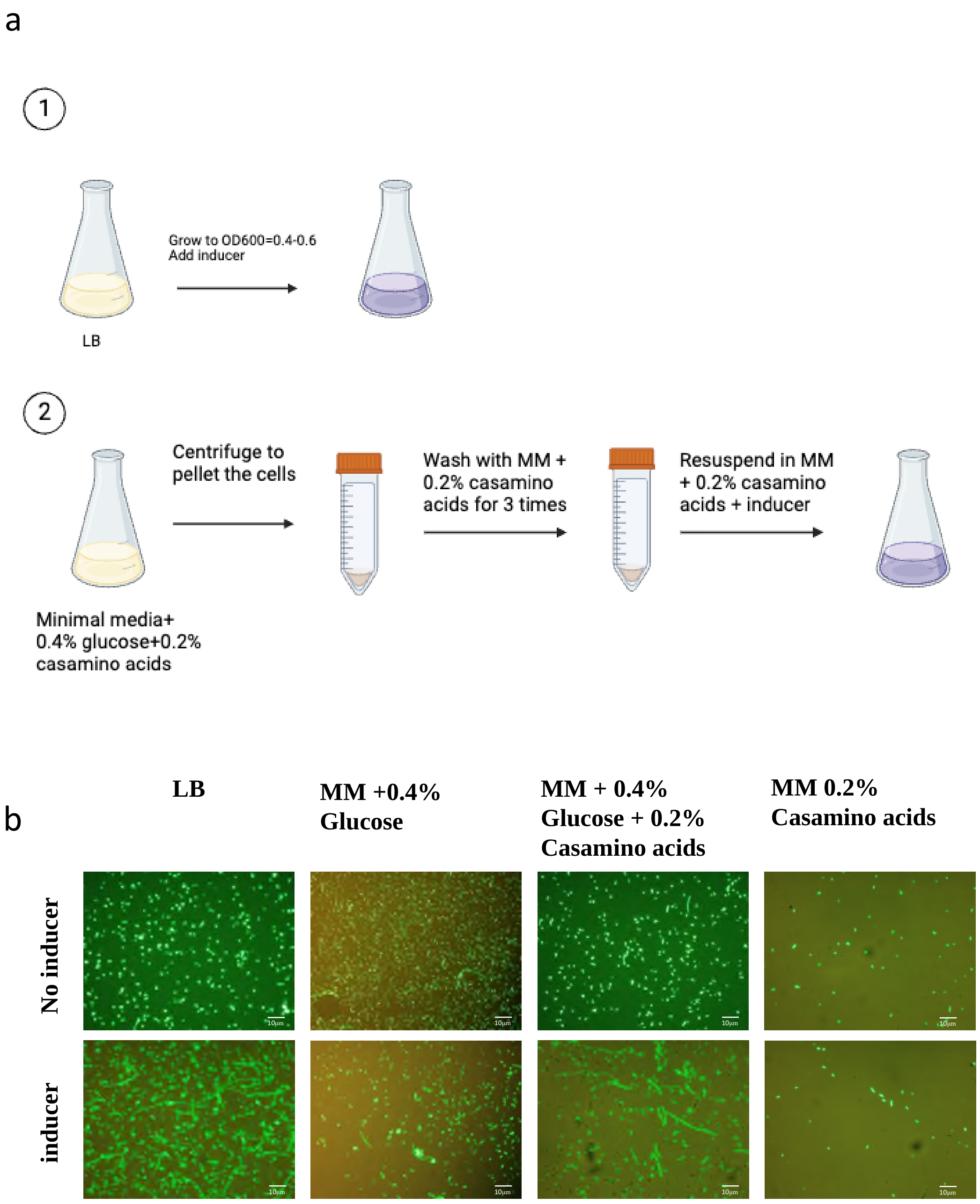

Supplementary Figure S2. Purification procedure and characterisation for ClearColi SimCells (a) Schematics of the purification workflow for ClearColi SimCell conversion utilizing either LB media or minimal media exchange to yield SimCells of high purity and uniform sizes. **(b)** Fluorescence microscopic images of the ClearColi cells before and after

induction into SimCells, using different media types. The magnification is 20x, with a scale bar of 10µm presented for reference.

Supplementary Figure S3

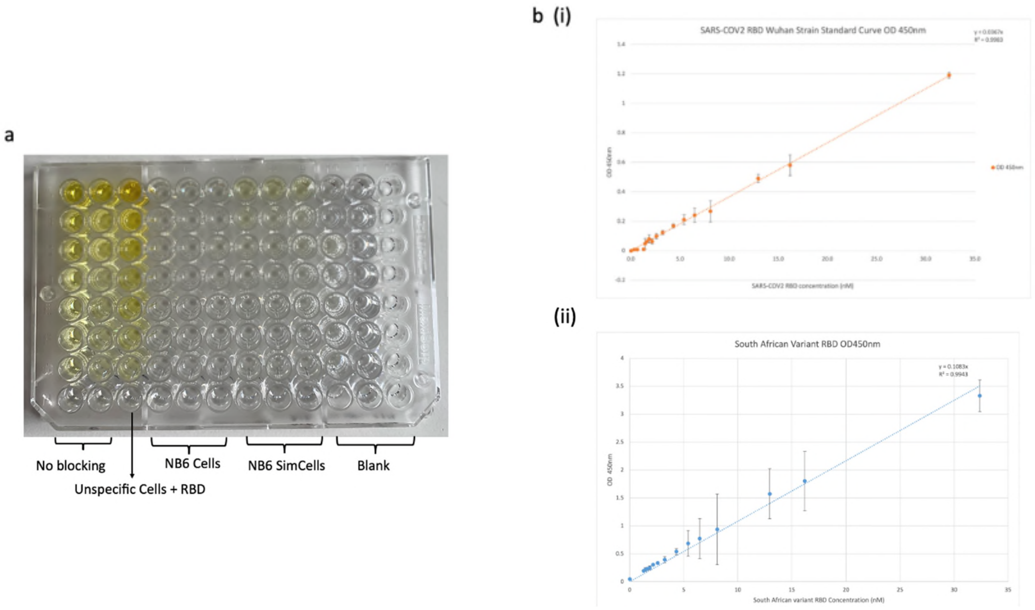

Supplementary Figure S3. (a) Top-down image of pNV_Nb6 sfGFP whole-cell and pNV_Nb6 sfGFP SimCell neutralization assay plate. The neutralization assay was repeated for three biological replicates. **(b) (i)** Calibration standard curve for Wuhan HRP-RBD. The equation of the standard curve is displayed in the top-right corner of the graph: y (OD450nm) = 0.0367 x (HRP-RBD concentration). **(ii)** Calibration standard curve for the South African variant HRP-RBD. The equation of the standard curve is displayed in the top-right corner of the graph: y (OD450nm) = 0.1083 x (HRP-RBD concentration).

Supplementary Figure S4

a

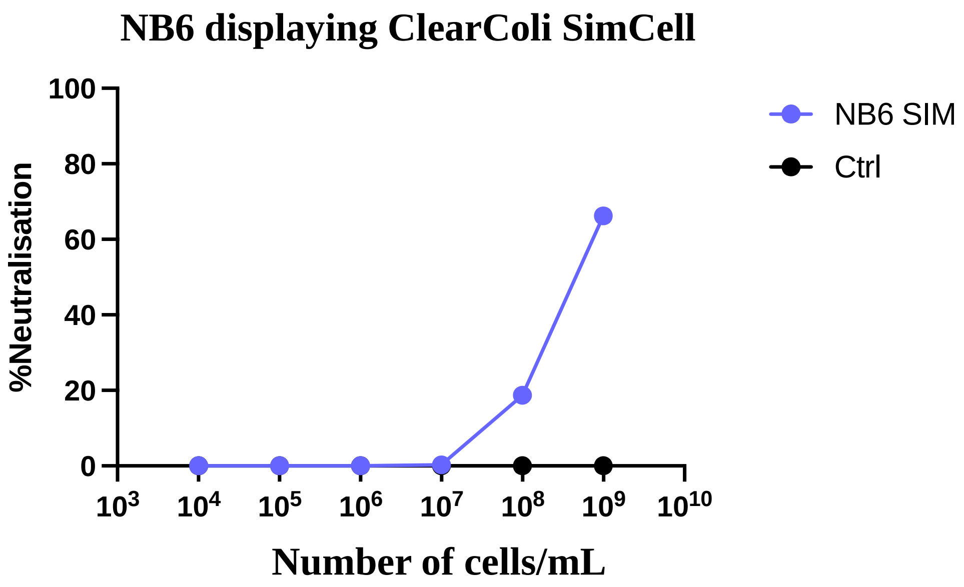

b

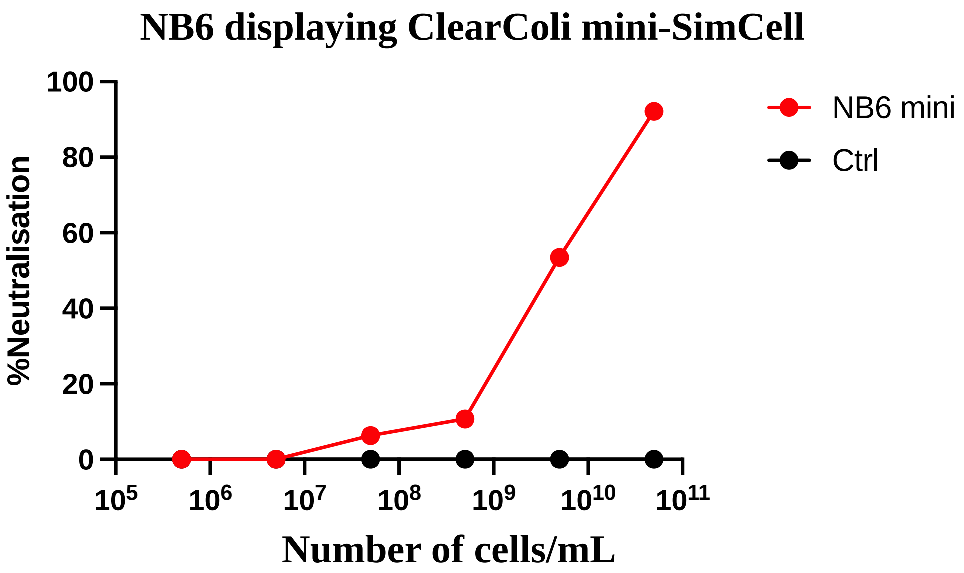

a

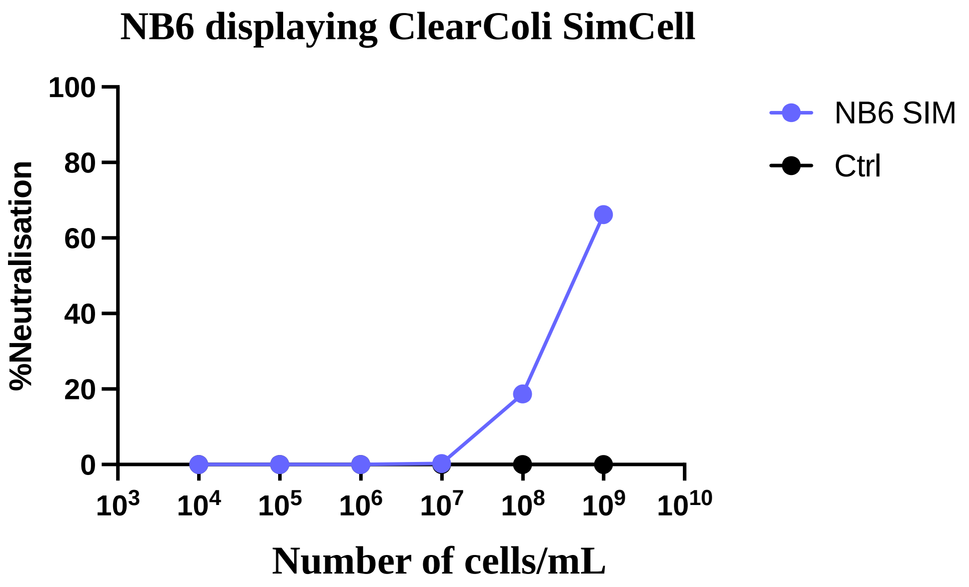
NB6 displaying ClearColi SimCell
%Neutralisation
100
80
60
40
20
0
10^3 10^4 10^5 10^6 10^7 10^8 10^9 10^{10}
Number of cells/mL
NB6 SIM
Ctrl

b

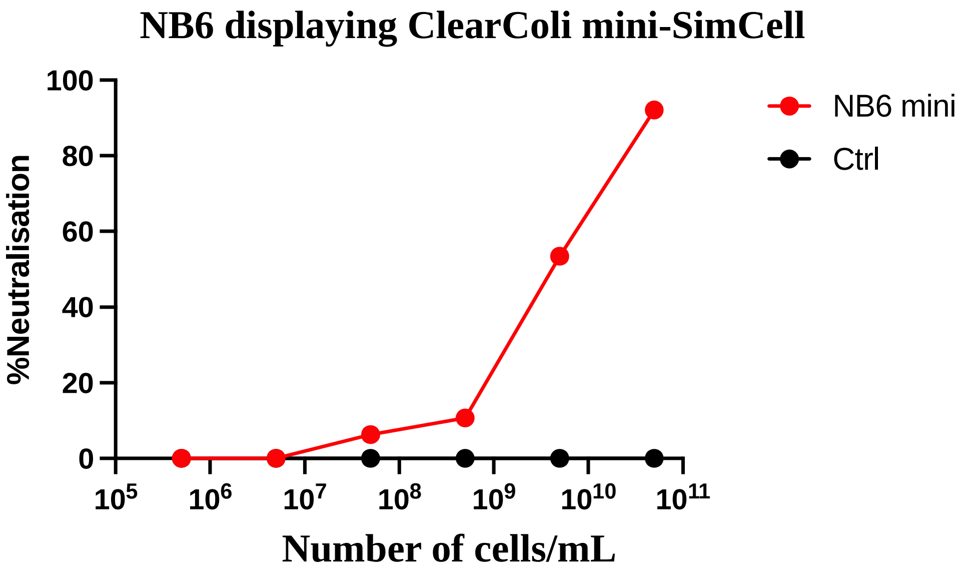
NB6 displaying ClearColi mini-SimCell
%Neutralisation
100
80
60
40
20
0
10^5 10^6 10^7 10^8 10^9 10^{10} 10^{11}
Number of cells/mL
NB6 mini
Ctrl

c

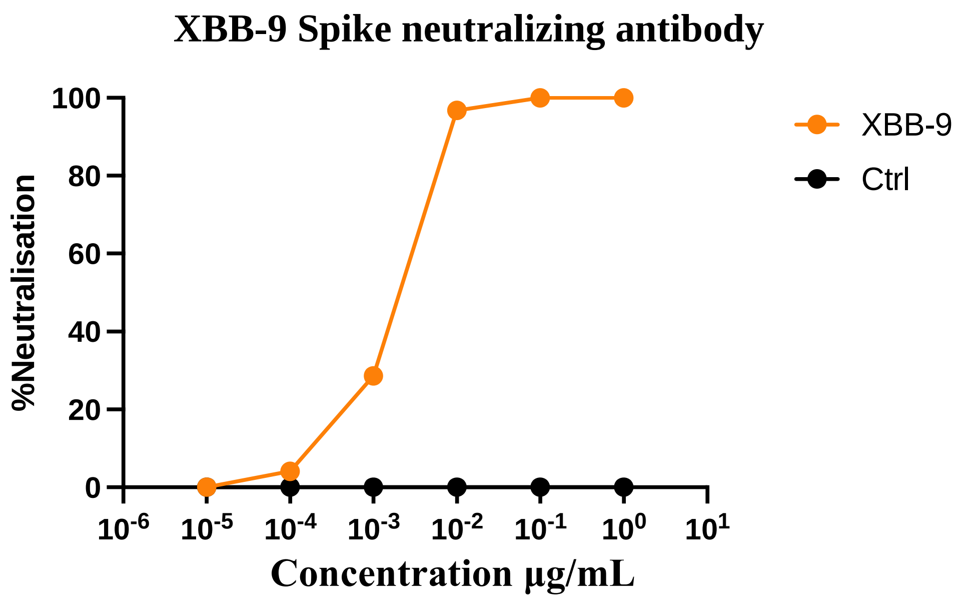

Supplementary Figure S4. Neutralization curves of anti-S RBD nanobody NB6 displaying SimCell and mini-SimCell. This panel illustrates the neutralization curves of **(a)**NB6 anti-Spike RBD monomeric nanobody-expressing ClearColi SimCell and **(b)** mini-SimCells, and with non-binding (anti-HER2) nanobody-expressing SimCell/mini-SimCells serving as a negative control, against the Victoria and B.1.351 (Beta) variants. SimCells were serially diluted from 10^9/mL to 10^4/mL. Mini-SimCells underwent a 10-fold serial dilution five times, starting from 5×10^10/mL to 5×10^5/mL. For the assay, 50µL of SimCell/mini-SimCell samples were mixed with 200 viral foci in 25µL per well. **(c)** neutralization curves of the XBB-9 antibody, known for its neutralization capability against both the Victoria and Beta variants, was used as a positive control.

Reference:

1. Mamat, U. *et al.* Endotoxin-free protein production-ClearColi™ technology. (2013) doi:10.1038/nmeth.f.367.
2. Hanke, L. *et al.* An alpaca nanobody neutralizes SARS-CoV-2 by blocking receptor interaction. *Nat Commun* **11**, 1–9 (2020).
3. Schoof, M. *et al.* An ultrapotent synthetic nanobody neutralizes SARS-CoV-2 by stabilizing inactive Spike. *Science (1979)* **370**, 1473–1479 (2020).
4. Esparza, T. J., Martin, N. P., Anderson, G. P., Goldman, E. R. & Brody, D. L. High affinity nanobodies block SARS-CoV-2 spike receptor binding domain interaction with human angiotensin converting enzyme. *Sci Rep* **10**, 1–13 (2020).
